# Nephroprotective effects of *Datura stramonium* leaves against methotrexate nephrotoxicity via attenuation of oxidative stress-mediated inflammation and apoptosis in rats

**DOI:** 10.22038/AJP.2023.21903

**Published:** 2023

**Authors:** Esther U Alum, Ademola C. Famurewa, Obasi U Orji, Patrick M Aja, Felix Nwite, Simon E Ohuche, Stanley C Ukasoanya, Lucy O Nnaji, Deborah Joshua, Kingsley U Igwe, Stephen F Chima

**Affiliations:** 1 *Department of Biochemistry, Faculty of Science, Ebonyi State University, PMB, 053, Abakaliki, Nigeria*; 2 *Department of Medical Biochemistry, Faculty of Basic Medical Sciences, College of Medical Sciences, Alex Ekwueme Federal University, Ndufu-Alike, Ikwo, Ebonyi State, Nigeria*; 3 *Department of Pharmacology, Manipal College of Pharmaceutical Sciences, Manipal Academy of Higher Education, Manipal, Karnataka State, India*

**Keywords:** Methotrexate, Inflammation, Antioxidants, Apoptosis, D. stramonium

## Abstract

**Objective:**

Methotrexate (MTX) is a frontline antimetabolite anticancer drug which is used in different cancer treatments but its nephrotoxicity is a notable drawback that limits its clinical use. The present study was undertaken to examine whether *Datura stramonium* leaf extract (DSLE) could block MTX nephrotoxic side effect in rats.

**Materials and Methods:**

Animals were divided randomly into Control, Ethanol extract, MTX, and Extract + MTX groups. DSLE (200 mg/kg bw) was orally administered for 21 days, while MTX was injected intraperitoneally (ip) on the 18^th^ day. Serum levels of urea, creatinine and uric acid were determined. Kidney samples were used to determine glutathione peroxidase (GPx), superoxide dismutase (SOD), and catalase (CAT) activities, and renal levels of malondialdehyde (MDA), reduced glutathione (GSH), nitric oxide (NO), interleukin-6 (IL-6), tumor necrosis factor-α (TNF-α) and caspase-3.

**Results:**

Injection of MTX resulted in considerable increases (p<0.05) in creatinine, urea, and uric acid levels as well as renal MDA, NO, IL-6, TNF-α and caspase-3 compared to the controls. SOD and GPx increased significantly, while GSH was significantly depleted. Interestingly, DSLE markedly reduced (p<0.05) levels of creatinine, urea, uric acid, TNF-α, NO, MDA and caspase-3, whereas renal GSH increased markedly compared to the MTX group.

**Conclusion:**

DSLE has nephroprotective activity against MTX toxicity. However, further mechanistic studies are needed.

## Introduction

Anticancer chemotherapy mainly targets cancer cell DNA and replication machinery to induce DNA damage, cell cycle arrest and apoptosis. Chemotherapy is advantageous because the intravenous administration distributes the cytotoxic drug to the systemic tissues and so can kill cancer cells and prevent metastases (Ashrafizadeh et al., 2020). However, the disadvantages of cytotoxic anticancer drugs are unpleasant side effects and chemoresistance (Afsar et al., 2021). Methotrexate (MTX) is an antimetabolite frequently applied in the treatment of several cancers and non-neoplastic diseases such as autoimmune diseases (Hassanein et al., 2021; Famurewa et al., 2019). The anticancer action mechanism of MTX is consistent with the enzymatic inhibition of conversion of dihydrofolic acid to tetrahydrofolic acid needed for the synthesis of thymine to be incorporated into DNA. By implication, MTX thus inhibits synthesis of nucleotide and DNA in rapidly dividing cells. The inhibition results in apoptosis and cell cycle S phase arrest in cancer cells (Famurewa et al., 2019; Bordbar et al., 2018). However, the clinical use of MTX is often limited owing to its toxic influence on different critical tissues of the body. This is because of its double-edged action on cancer cells and rapidly-dividing cells (Sayed et al., 2021). 

The kidney is an important target of MTX toxicity; however, hepatotoxicity, neurotoxicity, testicular and intestinal toxicities have also been associated with MTX (Hassanein et al., 2021; Sayed et al., 2021; Abdel-Wahab et al., 2020; Famurewa et al., 2019a). Consistent evidence exists in the literature that implicates the effect of oxidative stress, pro-inflammation, apoptosis and other intricate signaling pathways on the pathogenesis of MTX-induced nephrotoxicity (Ahmed et al., 2021; Hassanein et al., 2018; Mahmoud et al., 2018). Investigations have revealed that MTX promotes generation of free radicals in the kidney leading to renal oxidation, lipid peroxidation and diminution of renal antioxidant capacity, and nephrotoxicity (Sayed et al., 2021). Further, MTX-induced generation of nitric oxide NO via induced nitric oxide synthase activity upregulates oxidative signaling, inflammatory and apoptotic cascades (Khalifa et al., 2017). About 90% of MTX is cleared by the kidney from the circulation (Stark et al., 1989). Therefore, MTX can trigger renal dysfunction and elevated levels of serum creatinine and urea (Ahmed et al., 2021). 

Medicinal plants are a reservoir of bioactive compounds capable of counteracting chemotherapy-triggered toxic injuries (Mahmoud et al., 2018). *Datura stramonium* is a medicinal plant in the family of *Solanaceae *that grows in the tropic and temperate regions of the world; it originates from America but it is currently distributed around the world, including India, Australia, China, Ethiopia, and Nigeria (Céspedes-méndez et al., 2021; Melaku and Amare, 2020). *D. stramonium *is a leafy, erect, stout, smooth and pale yellow-green plant that grows to about 1.2 m high (Melaku and Amare, 2020). *D. stramonium *has demonstrated pharmacological efficacies such as anticancer, antioxidative, antiinflammatory, antiasthmatic antifungal and anticholinergic effects (Nasir et al., 2022; Sharma et al., 2021; Soni et al., 2012). *D. stramonium* seed and leaves could abrogate cyclophosphamide- and CCl_4_-induced oxidative inflammation (Nasir et al., 2022; Joshua et al., 2019). However, we have considered *D. stramonium* for this study because published evidence indicates its prowess as a deposit of antioxidant and antiinflammatory agents. In addition, to our current knowledge, there is no study on the effect of *D. stramonium* leaf extract (DSLE) on MTX-induced kidney damage. Thus, the current investigation was conceived to evaluate whether DSLE could prevent the MTX nephrotoxicity in rats. 

## Materials and Methods

**Chemicals and reagents**

The reagents and chemicals that were used for this study were of analytical grade. They were procured from May and Baker, BDH, England; and Merck, Darmstadt, Germany. The commercial kits were procured from Biosystem Reagents and Instruments, Spain and Randox, QCA, USA. 

**Biological materials**

*D. stramonium* leaves and the Wistar rats (male) were referred to as biological materials used. Fresh leaves of fully-grown *D. stramonium* were collected from Pastoral Centre, Abakaliki Town, Ebonyi State, Nigeria in October 2021 and identified by Mr. Nwankwo Onyebuchi, at the Department of Applied Biology Ebonyi State University, Abakaliki, Nigeria, and the voucher number generated was EBSU-H-397.

**Animal handling**

Wistar strain rats (95–105 g) purchased from the Animal Science Department, University of Nigeria, Nsukka, Enugu were used in this study. Before the study, rats were made to acclimatize for 2 weeks in the Animal House, Department of Biochemistry, Ebonyi State University, Abakaliki, at temperature 25±2°C and photoperiod 12 hr light and 12 hr dark. The rats were given commercial Vital Feeds Nigeria Ltd, Jos, Nigeria and were exposed to water *ad libitum*. The animal handling protocols were according to the procedures established by the NIH Publication (NIH Publication No. 85‐23, revised 1996) on Guide for the Care and Use of Laboratory Animals. In addition, the ethical approval of the Ethics Committee of Department of Biochemistry, Ebonyi State University, Abakaliki, Nigeria (EBSU-H-397) was obtained. 

***D. stramonium*** **ethanol leaf extract**

Oluduro and Aderiye (2009) method was used for ethanol extraction. The *D. stramonium* leaves were cleansed and dried in the shade and made to powder in a grinder and sifted using 0.25 mm sieve. The powdered sample was used for the extraction. Eight hundred grams (800 g) of the sample were soaked in 2000 ml of absolute ethanol for 48 hr at room temperature with intermittent shaking. Thereafter, the filtrate was obtained by sieve cloth and heated on a water bath at 35^o^C for significant removal of solvent. The extract was stored in airtight container. 

**Experimental design**

The animals were divided into 4 group after 14 days of acclimatization. Group 1 (Normal control): Rats were given 5 ml/kg normal saline oral gavage for 21 consecutive days. Group 2 (Extract): Rats were administered with *D. stramonium* leaf extract (DSLE) oral gavage at the dose of 200 mg/kg body weight for 21 days (Nasir et al., 2020). Group 3 (MTX): Rats were injected a single dose of 20 mg/kg body weight intraperitoneal (ip) on day 18 (Famurewa et al., 2017). Group 4 (Extract + MTX): Rats were administered with the extract by oral gavage (200 mg/kg bw) for 21 days + injection of MTX (20 mg/kg bw) ip on day 18 (Nasir et al., 2020; Famurewa et al., 2017)

After the treatment, overnight fasted animals were anaesthetized under mild diethyl ether anesthesia for collection of blood samples from the cardiac puncture. Whole blood was collected into plain bottles and centrifuged after 1hr to obtain serum samples. Rats were then sacrificed for kidney collection. The kidneys were cleaned in cold saline solution before separately homogenization in Bouin’s fluid (1:5 w/v, pH 6.4) and centrifugation at 3500 rpm for 20 min at 4°C. The kidney supernatant and serum obtained were used for biochemical parameters assessment in the current investigation. The renal samples were preserved in 10% buffered formalin for histological analysis.

**Determination of oxidative stress indices**

In the current study, rat ELISA kits purchased from BioDiagnostics, Giza, Egypt were used. Thiobarbituric acid reactive substance (TBARS) product expressed as malondialdehyde (MDA) was determined spectrophotometrically (Wallin et al., 1993). Nitric oxide (NO) level was estimated according to the method of Bories and Bories (1995) based on the Griess reaction. Reduced glutathione (GSH) was measured by the method of Ellman (1959). The activity of superoxide dismutase (SOD) was assessed by McCord and Fridovich (1969) method. Catalase activity was measured by the method of Sinha (1972). GPx activity was measured by Paglia and Valentine (1967).

**Determination of inflammatory cytokines**

The renal levels of TNF-α and IL-6 were estimated using RayBio ELISA kit (USA), according to the manufacturer’s label instructions. 

**Determination of renal indices**

Rat ELISA kits (Randox Chemicals, USA) were used for urea, creatinine and uric acid determination. The level of serum creatinine was assessed by alkaline picrate Henry et al. (1974) method. The level of urea was determined according to modified diacetyl monoxime method of Wybenga et al. (1971). Uric acid was estimated according to Fossati et al. (1980) method based on the formation of allantoin and H_2_O_2_ by uricase activity. The products were converted by peroxidase to chromogen compound which was read at 520 nm.

**Histological analysis**

Samples of the kidney fixed in 10% formalin for 48 hr, were processed via ethanol dehydration and embedded in paraffin blocks. Block sectioning (5 µm) was carried out by microtome followed by setting on slides for hematoxylin and eosin (H&E) staining. The prepared slides were viewed under a light microscope.

**Data analysis**

Results are described as mean±standard deviation (SD). Data were analyzed and compared using ANOVA followed by turkey post hoc test. The results were considered significant at p<0.05. All statistical analysis was carried out using Graph Pad Prism version 5.00 for Windows (GraphPad Company, San Diego, USA). 

## Results

**Effect of DSLE on renal function indices in MTX-induced nephrotoxicity**

**Figure 1:**
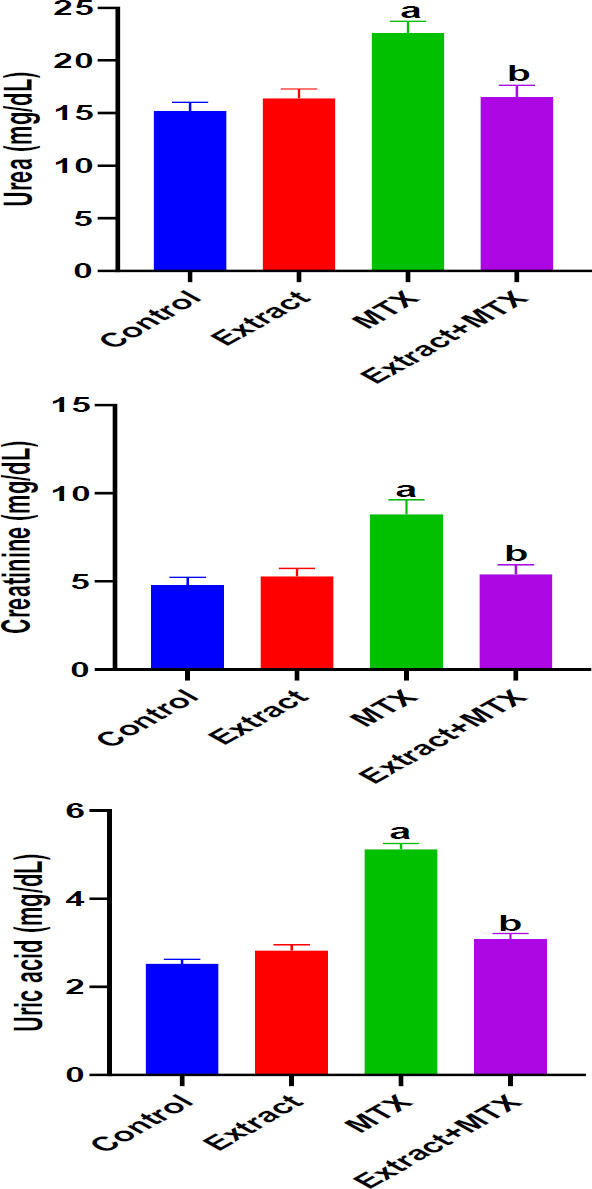
Effect of extract and MTX (methotrexate) on serum urea, creatinine and uric acid levels in MTX-induced nephrotoxicity. Data are expressed as mean±SD. ^a^p<0.05 indicates significant difference compared to the normal control group. ^b^p<0.05 indicates significant difference compared to the MTX group.

Figure 1 depicts the effect of DSLE and MTX on serum urea, creatinine and uric acid in rats injected with MTX. MTX caused significantly (p<0.05) increased levels of serum urea, creatinine and uric acid compared to the normal control group. On the other hand, administration of DSLE to rats resulted in marked (p<0.05) decreases in levels of serum urea, creatinine and uric acid compared to the MTX group. 

**Effect of DSLE on renal GSH and MDA in MTX-induced nephrotoxicity**

**Figure 2:**
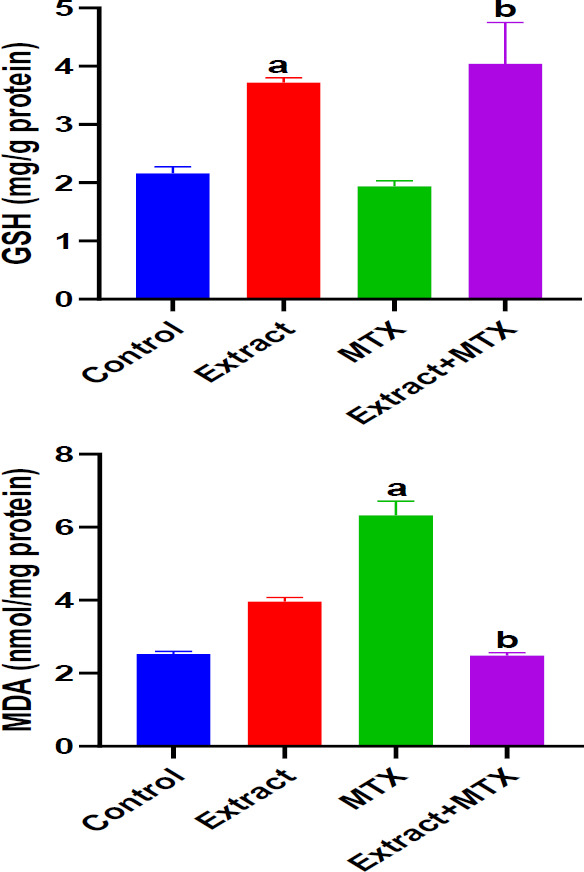
Effect of DSLE and MTX (methotrexate) on renal levels of glutathione (GSH) and malondialdehyde (MDA) in MTX-induced nephrotoxicity. Data are expressed as mean±SD. ^a^p<0.05 indicates a significant difference compared to the normal control group. ^b^p<0.05 indicates a significant difference compared to the MTX group.

Figure 2 presents the effect of extract and MTX on renal levels of GSH and MDA in rats injected with MTX. MTX caused a significant (p<0.05) decrease in GSH level, whereas the level of MDA increased considerably compared to the normal control group. On the other hand, administration of DSLE to rats resulted in a marked (p<0.05) increase in GSH levels and a decrease in MDA compared to the MTX group.

**Effect of DSLE on renal SOD, GPx and CAT in MTX-induced nephrotoxicity**

**Figure 3:**
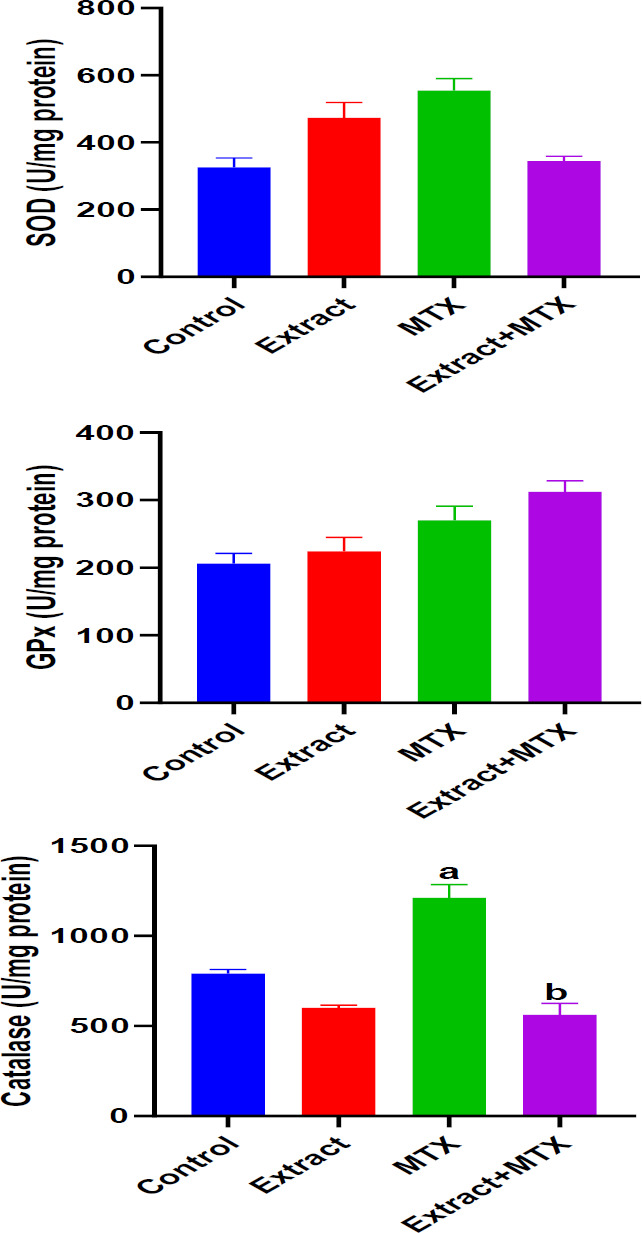
Effect of DSLE and MTX (methotrexate) on renal activities of superoxide dismutase (SOD), glutathione peroxidase (GPx) and catalase in MTX-induced nephrotoxicity. Data are expressed as mean±SD. ^a^p<0.05 indicates significant difference compared to the normal control group. bp<0.05 indicates significant difference compared to the MTX group.

Figure 3 presents the effect of extract and MTX on renal activities of antioxidant enzymes, SOD, GPx and catalase (CAT) in rats injected with MTX. MTX caused insignificant (p>0.05) increases in the activities of SOD and GPx, as well as significant (p<0.05) increases in CAT compared to the normal control group. On the other hand, administration of DSLE to rats only resulted in an insignificant (p>0.05) increase in GPx activities compared to the MTX group.

**Effect of DSLE on renal inflammation and caspase-3 in MTX-induced nephrotoxicity**

**Figure 4:**
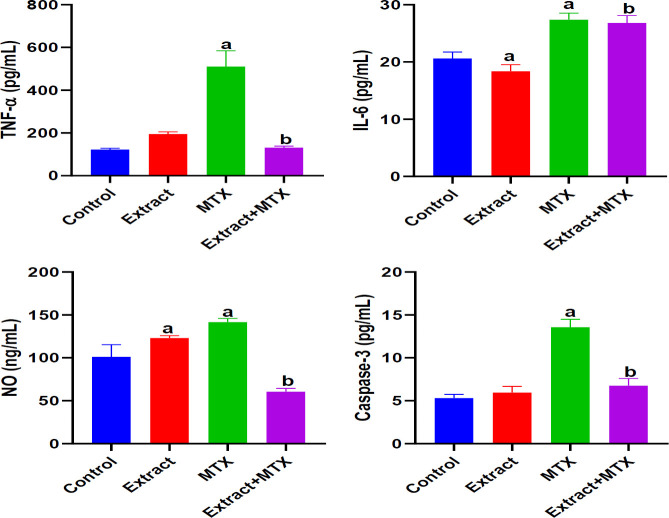
Effect of extract and MTX (methotrexate) on renal levels of TNF-α (tumor necrosis factor-alpha), IL-6 (interleukin-6), NO (nitric oxide) and caspase-3 in MTX-induced nephrotoxicity. Data are expressed as mean±SD. ^a^p<0.05 indicates significant difference compared to the normal control group. ^b^p<0.05 indicates significant difference compared to the MTX group.

Figure 4 presents the effect of DSLE and MTX on renal levels of TNF-α, IL-6, NO and caspase-3 in rats injected with MTX. MTX induced significant (p<0.05) increases in TNF-α, IL-6, NO and caspase-3 levels compared to the normal control group. On the other hand, administration of DSLE to rats exerted considerable decreases in TNF-α, NO and caspase-3 levels compared to the MTX group. 

**Effect of DSLE on renal histology of MTX-intoxicated rats**

**Figure 5:**
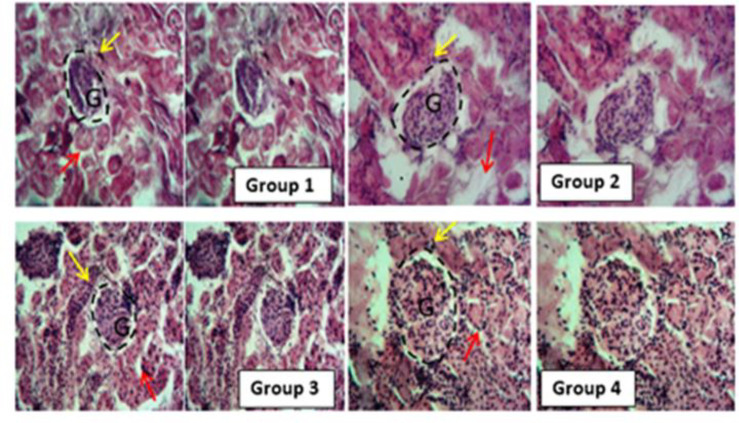
Effect of DSLE and MTX on histological architecture of kidneys in MTX-induced nephrotoxicity. Groups 1 and 2 (Normal control and Extract, respectively): showing normal renal corpuscle with typical renal histology (red arrow: convoluted tubules, yellow arrow: podocytes, with dotted lines indicating G for glomerulus and glomerular capsule space. Group 3 (MTX): G indicates atrophied glomerulus with collapsing space, non-distinct necrotic tubular cells with vacuolated cytoplasm (arrows). Group 4 (Extract + MTX): We observed mild degeneration, ameliorated necrosis but distinct glomerulus near group 1 and 2.

Figure 5 shows the histological view of renal architecture in MTX-intoxicated rats. In normal control and Extract groups (group 1 and 2), the histology revealed normal glomerulus, podocytes, and tubules. In MTX (group 3) however, the MTX injection caused alterations consistent with necrotic tubules, atrophied glomerulus (arrows). Extract +MTX (Group 4) showed mild alterations and recovery of structures from MTX damage. 

## Discussion

Methotrexate is an antifolate anticancer drug used in the treatment of non-cancer diseases such as rheumatoid arthritis, psoriasis, and graft rejection disease (Kelleni et al., 2016). Its use during chemotherapy exerts hepatotoxicity via the mechanism of oxidative stress, pro-inflammation and apoptotic cascades (Famurewa et al., 2019b). Natural plants products are known as inhibitors and modulators of apoptosis, inflammation and free radical effects (Prasanna et al., 2020; Moghaddam et al., 2020). Thus, we have assayed whether the natural extract of *D. stramonium* leaves would prevent MTX-induced nephrotoxicity in a rat model. 

In this investigation, the results portray that the antineoplastic drug, MTX at the dose 20 mg/kg induced nephrotoxic injury via oxidative stress. The renal damage was shown via the marked increases in renal MDA levels and serum levels of uric acid, creatinine and urea, and considerable decreases in renal GSH (Figures 1 and 2 ▶). Our observation of increased levels of MDA is a biochemical indication of MTX-induced oxidative stress and generation of free radicals (Famurewa et al., 2017; Fakurazi et al., 2012). Literature shows that oxidative stress leads to renal damage that could be manifested as depressed renal function and consequent increases in uric acid, creatinine and urea levels (Famurewa et al., 2017). It could therefore be conceived in this study that MTX triggered oxidative renal degeneration and dysfunction, leading to altered levels of MDA, GSH and elevated serum uric acid, creatinine and urea level. The histological degeneration found in this study is a confirmation of the biochemical alterations (Figure 5). These findings are consistent with earlier reports that MTX injection at 20 mg/kg could trigger renal dysfunction (Hassanein et al., 2021 Mahmoud et al., 2018). Intriguingly, the results of our assays for further evaluation of antioxidant enzymes revealed increased SOD, CAT and GPx activities in rats injected with MTX only (Figure 3). But this is contrary to the study reports in the existing literature (Hassanein et al., 2018; Mahmoud et al., 2018). However, certain earlier studies report the possible increases in antioxidant enzyme activities as a protective compensatory mechanism against cell damage (Vardi et al., 2008; Uzar et al., 2006). According to Miyazono et al. (2004) and Uzar et al. (2006), MTX caused prominent increases in CAT and SOD activities in the cerebellum and small intestine of rats, corroborating our findings herein. Vardi et al. (2008) further explain that moderate cellular exposure to toxicants may elevate gene expressions of antioxidant apparatus, while high level exposure to toxicants could lead to depression of antioxidant mechanism. Perhaps the exposure of the kidney to MTX attack triggered increased activities of CAT, SOD and GPx as a compensatory defense in our study herein. Nevertheless, the elevated MDA and depression of GSH, in part, indicate *de novo *induction of oxidative stress by MTX, and the oxidative damage might have caused the renal dysfunction underscored by increased levels of serum creatinine, urea and uric acid. Interestingly, the administered DSLE prevented kidney damage and significantly reduced the levels of serum creatinine, urea and uric acid with corresponding improvements in renal levels of MDA and GSH. These results demonstrate antioxidant nephroprotective effect of DSLE against MTX-triggered renal damage or dysfunction. Previous studies have shown that DSLE could improve renal status against drug toxicity. For example, in the study of Joshua et al. (2019), it was found that DSLE markedly improved levels of MDA, GSH, creatinine and urea against cyclophosphamide-induced renal damage. In fact, the very close species to *Datura stramonium*, *Datura metel*, has also shown no toxicity on the kidney (Imo et al., 2019) and antioxidant nephroprotective efficacy against gentamicin drug (Alam et al., 2020). However, the observed beneficial effect of DSLE may be associated with its contents. It is widely published that DSLE phytochemical analysis shows health-promoting bioactive alkaloids, such as gallic acid, catechin, rutin, apigenin and other bioactive compounds (Yadav et al., 2021; Nasir et al., 2020; Soni et al., 2012). Conceivably, these compounds might be contributing to the nephroprotective effect of DSLE observed in the current study. 

Methotrexate is well published to induce inflammatory cascades in humans and animal models (Ezhilarasan, 2021). It has been shown that oxidative stress induces inflammation which often leads to apoptotic signaling (Ahmed et al., 2021; Abdel-Wahab et al., 2020). To this end, we thus explored the role of pro-inflammatory markers and caspase-3 in the current study. In agreement with the previous findings, we observed that MTX injection promoted the expression of cytokines and apoptosis (Figure 4). The renal level of pro-inflammatory markers, TNF-α, IL-6, NO and apoptotic caspase-3 were significantly elevated in comparison to the control group levels. These results indicate the pro-inflammatory nature of MTX. At the molecular level, MTX has been indicated to cause free radical generation which triggers the activation of oxidative pro-inflammation via increased activities of enzymatic pro-inflammatory modulators, such as cyclooxygenase-2 (COX-2), inducible nitric oxide synthase (iNOS) and cytokines- IL-1β, TNF-α, and IL-6 (Khafaga and El-Sayed, 2018). The studies of Mahmoud et al. (2018) and Hassanein et al. (2018) show that MTX increased the kidney levels of TNF-α, NO and apoptosis markers in agreement with our findings in this study. Although our study did not analyze iNOS, the increased level of NO in the kidney in our study is a surrogate reporter of increased activity of iNOS. During inflammation, synthesis of NO is significantly elevated beyond the physiological NO level by many folds, contributing to reactive oxygen species ROS-mediated tissue damage. Besides, the increased level of IL‐6 could induce further the iNOS and COX-2 production, which further unleash oxidative damage on the renal cells (Zhang et al., 2020). Caspase-3 is a key pro-apoptotic mediator and an indicator of irreversible activation of apoptosis (Ahmed et al., 2021; Khalifa et al., 2017). The increased renal level of caspase-3 found in our study has been earlier reported (Ahmed et al., 2021; Khalifa et al., 2017). Concisely, MTX induced pro-inflammation and apoptosis in the current study. However, we found the DSLE-induced downregulation of TNF-α, NO and caspase-3 in comparison to the MTX group. It is striking that the level of IL-6 failed to respond to the administration of DSLE. The reason for this observation is currently unclear within the context of this study. The bioactive contents of DSLE could as well combat the proinflammation and apoptosis ameliorating their renal levels and damage as observed in the histology result (Figure 5) (Nasir et al., 2022). Our findings agree with the report of Nasir et al. (2022) on the anti-inflammatory effect of DSLE *in vivo* and *in vitro* (Gupta et al., 2010). Therefore, our findings suggest apoptotic and inflammatory inhibitory actions of DSLE against MTX-orchestrated oxidative stress-mediated nephrotoxic injury in this study.

Taken together, DSLE represents a promising nephroprotective extract against MTX-induced nephrotoxicity and histological alterations. The nephroprotective effect of DSLE is related to its potential to ameliorate renal oxidative stress, inflammation and caspase-3 apoptosis induced by MTX. Nevertheless, future studies are undoubtedly needed for fractionation and isolation of beneficial compounds in DSLE for mechanistic investigations and possible drug discovery. 

## Conflicts of interest

The authors have declared that there is no conflict of interest.
